# Quality of observational studies of clinical interventions: a meta-epidemiological review

**DOI:** 10.1186/s12874-022-01797-1

**Published:** 2022-12-07

**Authors:** Sergei Grosman, Ian A. Scott

**Affiliations:** 1 Department of Internal Medicine and Clinical Epidemiology, Princess Alexandra Hospital, 199 Ipswich Road, Brisbane, Queensland 4102 Australia; 2 Department of Medicine, Cairns Hospital, 165 The Esplanade, Cairns, Queensland 4870 Australia

**Keywords:** Observational studies, Methodology, Quality, Case series

## Abstract

**Background:**

This meta-epidemiological study aimed to assess methodological quality of a sample of contemporary non-randomised clinical studies of clinical interventions.

**Methods:**

This was a cross-sectional study of observational studies published between January 1, 2012 and December 31, 2018. Studies were identified in PubMed using search terms ‘association’, ‘observational,’ ‘non-randomised’ ‘comparative effectiveness’ within titles or abstracts. Each study was appraised against 35 quality criteria by two authors independently, with each criterion rated fully, partially or not satisfied. These quality criteria were grouped into 6 categories: justification for observational design (*n* = 2); minimisation of bias in study design and data collection (*n* = 11); use of appropriate methods to create comparable groups (*n* = 6); appropriate adjustment of observed effects (*n* = 5); validation of observed effects (*n* = 9); and authors interpretations (*n* = 2).

**Results:**

Of 50 unique studies, 49 (98%) were published in two US general medical journals. No study fully satisfied all applicable criteria; the mean (+/−SD) proportion of applicable criteria fully satisfied across all studies was 72% (+/− 10%). The categories of quality criteria demonstrating the lowest proportions of fully satisfied criteria were measures used to adjust observed effects (criteria 20, 23, 24) and validate observed effects (criteria 25, 27, 33). Criteria associated with ≤50% of full satisfaction across studies, where applicable, comprised: imputation methods to account for missing data (50%); justification for not performing an RCT (42%); interaction analyses in identifying independent prognostic factors potentially influencing intervention effects (42%); use of statistical correction to minimise type 1 error in multiple outcome analyses (33%); clinically significant effect sizes (30%); residual bias analyses for unmeasured or unknown confounders (14%); and falsification tests for residual confounding (8%). The proportions of fully satisfied criteria did not change over time.

**Conclusions:**

Recently published observational studies fail to fully satisfy more than one in four quality criteria. Criteria that were not or only partially satisfied were identified which serve as remediable targets for researchers and journal editors.

**Supplementary Information:**

The online version contains supplementary material available at 10.1186/s12874-022-01797-1.

## Introduction

The growth of electronic medical records and other ‘real-world’ digitised sources of clinical data has led to a proliferation of observational studies of the effectiveness of clinical interventions. While the scientific standard for assessing intervention efficacy remains randomised controlled trials (RCTs), well-designed observational studies have been used to elucidate potential harms, and expand the evidence base in situations where existing RCTs have limited generalisability because of selective patient enrolment or outcome reporting, or new RCTs are logistically very difficult to perform [1]. The main concern with observational studies is their vulnerability to bias, particularly confounding by indication [2], whereby patients receive a therapy based on certain patient or clinician characteristics which may not be explicitly stated or recorded, but which are prognostically important and influence the outcome of interest, independently of the therapy [3]. In the past, influential observational studies have helped institutionalise scores of clinical practices for decades that were subsequently shown to be ineffective or indeed harmful when subjected to RCTs where randomisation eliminated selection bias in who received the experimental therapy [4].

Nevertheless, reviews of observational studies suggest that they often report effects and generate inferences similar to those of RCTs studying the same therapy and involving similar populations and outcome measures [5–7]. Advances in study design, statistical methods and clinical informatics have potential to lend greater rigour to observational studies [1]. Multiple guidelines detailing methodological [8] and reporting [9] standards, and instruments for assessing study quality [10–13] exist. Although systems for grading evidence quality, such as Grades of Recommendation, Assessment, Development and Evaluation (GRADE), rank observational studies as being of lower quality than RCTs, they can be regarded as sources of valid data if they are well designed, show large effect sizes and account for all plausible confounders [14]. Many systematic reviews include both RCT and high-quality observational studies in their analyses in deriving causal inferences [15].

However, the level of trustworthiness of observational studies remains controversial. We hypothesised that, due to advances in observational research, such studies are becoming more rigorous and valid. The aim of this meta-epidemiological study was to assess the methodological quality of a sample of recently reported non-randomised clinical studies of commonly used clinical interventions, and ascertain if quality is improving over time.

## Methods

In reporting this study, we applied the guidelines for meta-epidemiological methodology research proposed by Murad and Young [16]. No a priori study protocol existed or was registered.

### Study selection

A backward search from December 31, 2018 to January 1, 2012 was performed using PubMed with no language filters to identify observational studies of therapeutic interventions. Search terms comprised ‘association’, ‘observational,’ or ‘non-randomised’ or ‘comparative effectiveness’ within titles or abstracts. We included studies which: involved clinician-mediated therapeutic interventions administered directly to adult patients; reported comparison of two concurrent therapeutic interventions which could include ‘usual care’; and whose outcomes included patient-important sentinel events (ie mortality, serious morbid events, hospitalisations) rather than solely disease or symptom control measures.

We excluded studies that: 1) featured case control comparisons, historical controls only, single arm cohorts, adjunct therapies, diagnostic tests (with no associated therapeutic intervention) or cost-effectiveness analyses (with no separate comparative effectiveness data); 2) compared a single intervention group with a placebo group; 3) comprised RCTs, or reviews and meta-analyses of either RCTs or observational studies; 4) involved paediatric, anaesthetic or psychiatric interventions or patients; 5) analysed therapies which were highly specialised, or not in common use (eg genetically guided therapies, investigational agents in research settings); 6) assessed effects of system-related innovations rather than direct clinical care (eg funding or governance structures); 7) studied non-medical interventions (eg effects on cardiovascular outcomes of reducing salt consumption or increasing physical activity); 8) studied exposures, risk factors or prognostic factors that may influence therapeutic effectiveness but did not involve head to head comparisons of two interventions (eg effects of dose changes or co-interventions); or 9) were descriptive studies with no reporting of outcome measures. One author (SG) performed the search and initial study selection, with subsequent independent review by the second author (IAS).

### Data collection

From each selected study we extracted the following data: study title, journal, and date of publication; rationale stated in the introduction for choosing an observational study design; existence of a pre-specified study protocol; patient selection criteria; methods of data collection from primary sources; reference to validation checks for coded administrative data or longitudinal data linkage processes; methods for minimising recording bias (in administrative data), recall bias, social desirability bias, and surveillance bias (in clinical registry data); methods for assessing clinical outcomes; choice of predictor variables; population characteristics and statistical methods used for balancing populations; imputation methods used for missing data; subgroup analyses and interaction testing for identifying independent prognostic variables; use of unplanned post-hoc analyses; statistical methods used for adjusting for multiple outcome analyses, clustering effects (in multicentre studies) and time-dependent bias; effect size and confidence intervals; sensitivity analyses for unmeasured confounders; stated intervention mechanism of action, temporal relation between intervention and outcomes, and dose-response relationships; any falsification tests performed; comparisons with results of other similar studies; and statements about study limitations and implications for clinical practice.

### Application of quality criteria

Both authors independently read the full text articles of included studies and applied to each study a list of 35 quality criteria which were, with some modification, based on those the authors have previously published (Table 1) [17] and which covered criteria listed in previously cited critical appraisal and reporting guidelines for observational studies [10–13]. These quality criteria were grouped into 6 categories: justification for observational design (*n* = 2); minimisation of bias in study design and data collection (*n* = 11); use of appropriate methods to create comparable groups (*n* = 6); appropriate adjustment of observed effects (*n* = 5); validation of observed effects (*n* = 9); and authors interpretations (*n* = 2).

**Table 1:** Quality criteria used for assessing observational studies of interventions

Criteria	Explanations
Justification for observational design 1. RCTs non-existent or inadequate 2. RCTs not feasible	Given that RCTs have minimal vulnerability to bias, authors should provide, at the outset, reasons why they chose to perform an observational study rather than an RCT
Minimisation of bias in study design and data collection 3. Pre-specified study protocol 4. Clearly stated patient selection criteria 5. Representative study population 6. Prospective and verifiable data collection 7. Validation checks for coded administrative data 8. Validation checks for longitudinal data linkage processes 9. Minimisation of recording bias in administrative data 10. Minimisation of recall bias 11. Minimisation of social desirability bias 12. Minimisation of surveillance bias in clinical registry data 13. Independent assessment of outcomes	Observational studies should emulate RCTs and pre-specify all aspects of the intended study. Source and selection of the study population should be inclusive Study sample should be representative of all patients in whom the intervention may be used. Observational studies often use routinely collected clinical data that were not originally intended for research purposes and hence collected with less attention to validity or reliability. Data that is prospectively collected (ie in real time as care is provided, even if analysed later in retrospect) and capable of verification (ie data source is subject to curation and re-analysis) is more accurate. Data quality checks should include logic and range checks, level of agreement with random health record re-abstractions and ‘gold standard’ studies comparing data abstractions with standard clinical and laboratory criteria or expert panel review. Coded data in administrative datasets should specify the code assignment process and validation procedures for ensuring accurate ascertainment of diagnoses. Robust and validated data linkage processes need to be in place for collecting data on the same patients over time from different data sources. Recording bias can affect administrative datasets used for reimbursement purposes as a result of ‘up-coding’ (inclusion of all possible diagnoses and procedures) to maximise revenue. Recall bias occurs when exposures or outcomes are ascertained from self-reporting by patients who may selectively recall past events Social desirability bias occurs when patients may self-report outcomes that they expect clinicians will want to hear or are in accord with peer norms Surveillance (or detection) bias may affect clinical registries whereby outcome assessments occur more often than usual in persons receiving a particular intervention. Outcomes such as clinical events or should be adjudicated independently by clinical experts who are blind to intervention assignment. Mortality data needs to come from verifiable sources such as death registries.
Use of appropriate methods to create comparable groups 14. Appropriate statistical regression models for balancing populations 15. Model includes all important predictor variables 16. Appropriate selection and measurement of all important predictor variables 17. Majority of population sample included in analysis 18. Imputation methods for missing data 19. Comparison groups well balanced	Observational studies are prone to selection bias in clinician decision-making which may relate to patient factors (age, gender, diagnosis or disease severity, frailty, cognitive function, physical capacity, personal preferences), clinician factors (level of training or expertise) and system of care factors (supportive infrastructure). The intervention groups must be balanced in terms of their likelihood (or propensity) to have received the intervention under study and minimise confounding by indication. Several statistical techniques can be used to adjust for confounding, with propensity score based methods used most commonly. These scores define an individual’s ‘propensity’ or probability of receiving the intervention between 0 and 1, conditional on all factors likely to influence this decision (as above). Propensity scores are derived from regression models where intervention is the outcome (independent variable), and pre-intervention factors influencing whether patients receive the intervention are the predictors (dependent variables). The model should include all clinically relevant predictor variables as determined by clinical experts. A clinically valid rationale as to why these predictors were chosen, and the methods used to ascertain and measure them should be provided. These regression models should be applied to all or most of the study sample population to maintain study power Imputation methods should account for missing data, especially outcome data if the outcomes are infrequent (< 5% or 5 per 100 person years). The models should yield well balanced comparison groups as measured by standardised mean differences < 10% or variance ratios < 2.0.
Appropriate adjustment of observed effects 20. Subgroup analyses ad Interaction testing for identifying independent prognostic variables 21. Avoidance of unplanned post-hoc analysis 22. Correction for multiple outcome analyses 23. Adjustment for clustering effects in multicentre studies 24. Adjustment for time-dependent bias	The observed effects of the intervention should be subject to subgroup analyses and statistical interaction testing in identifying independent prognostic variables associated with greater or lesser intervention effects. Unplanned post-hoc analyses (or ‘data dredging’) which are not well justified should be avoided as they may be biased by researchers’ knowledge of main outcomes. The statistical significance of results of multiple analyses of several different outcomes should be corrected by appropriate methods (such as Bonferroni). Outcomes should be corrected for clustering effects if a study has been collected data from multiple sites where the interventions were being delivered, unless there is reasonable assurance that patients, clinicians, intervention mode and outcome measures were uniform across sites. Outcomes should be adjusted for time-varying co-variates (eg level of disease severity or timing of exposure to interventions) that may influence intervention effects and avoid immortal time bias and reverse causality.
*Validation of observed effects* 25. Large effect size 26. Exclusion of possible benefit in presence of negative results 27. Sensitivity analysis for unmeasured confounders 28. Plausibility of intervention mechanism of action 29. Temporal relation between intervention and outcomes 30. Dose-response relationship 31. Consistency with other studies of same intervention 32. Coherence with other studies of similar interventions 33. Falsification test for intervention effect specificity	Effect sizes should be reasonably large in presenting a high signal to noise ratio that provides a buffer to residual confounding. There is no validated threshold but we have chosen RR or OR ≤ 0.5 (see text). In studies which report a point estimate of no benefit or harm, the 95% confidence interval for the effect size should not cross over the line of unity, suggesting a possible benefit that the study was unable to uncover due to inadequate power, large numbers of drop-outs, or biases in data collection, patient selection, or analytic methods Sensitivity analyses should be performed to assess how prevalent and influential an unknown or unmeasured confounder would have to be to attenuate or annul the observed effect. Quantitative bias analysis or E value calculations are accepted methods. A cause and effect relationship between intervention and observed outcomes is more likely if they satisfy the following Bradford-Hill causality criteria: Plausible mechanism of action that explains how the intervention results in observed outcomes Credible temporal relation between when the intervention is implemented and the outcome observed Increasing therapeutic response with increasing intensity or dose of the intervention Consistency of results with those reported in other trials (randomised and non-randomised) of the same intervention in similar populations Coherence of results with those reported in trials of similar interventions Falsification (or effect specificity) test where manifestations of another disease condition on which the intervention will exert no plausible effect is compared between groups, with expected result of no difference between groups.
Authors’ interpretations 34. Study limitations acknowledged 35. Impartial statement of study implications	Given the vulnerability to bias of observational studies, authors should be totally candid and exhaustive in stating the limitations to their study, with particular emphasis given to selection bias in patient selection and adequacy of methods for balancing groups. For the same reasons, authors’ should interpret their findings cautiously, not overstate their significance or the implications for clinical practice, and indicate when their results should be confirmed by additional studies, in particular RCTs.

For each study, the extent to which each criterion was satisfied were categorised as fully satisfied (Y) – all elements met; partially satisfied (P) – some elements met; or not satisfied (N) – no elements met; or not applicable (NA) if that criterion was not relevant to the study design, analytic methods or outcome measures. Inter-rater agreement between authors for criterion categorisation was initially 95.2% and consensus was reached on all criteria after discussion.

### Summary measures and synthesis of results

For each study, we calculated the proportion of all applicable quality criteria categorised as fully, partially or not satisfied. For each criterion applicable at the level of individual studies, we calculated the proportion of studies which fell into each category of satisfaction. We calculated the proportion of criteria which were fully, partially or not satisfied by all studies for which criteria were applicable. Trend analysis assessed whether the proportion of applicable criteria that were fully, partially or not satisfied changed over time for studies published between 2012 and 2018. All analyses were performed using Excel functions or Graph Pad software.

## Results

### Study characteristics

The literature search identified 1076 articles from which 50 unique studies met selection criteria [18–67] of which 28 (56%) assessed non-procedural, mainly pharmacological, therapies [18–24, 26, 28, 31–33, 36, 38–40, 43, 44, 50, 53–56, 64–67], 15 (30%) assessed invasive procedures [27, 29, 30, 34, 35, 37, 41, 42, 51, 52, 58–63], 4 (8%) assessed investigational strategies [25, 45, 46, 57] and 3 (6%) assessed models of care [47–49]. Studies most frequently involved interventions related to cardiology (18/50; 36%) [19–21, 23, 26, 30, 32, 35, 39, 40, 44, 50, 52, 61, 64–67], surgery (13/50; 26%) [27, 29, 31, 34, 37, 38, 42, 53, 58–60, 62, 63], neurology (4/50; 8%) [28, 43, 49, 54] and oncology (4/50; 8%) [18, 25, 56, 57]. Most studies (36/50, 72%) [18–22, 24–32, 34–37, 40, 42–45, 48, 50, 52, 54, 55, 57–60, 63–65] were published in one journal (JAMA), with 13/50 (26%) [33, 38, 39, 41, 46, 47, 49, 51, 53, 56, 61, 62, 66, 67] in another (JAMA Internal Medicine). Sample size varied from as low as 464 participants [25] to as high as 1,256,725 [56]. Study characteristics are summarised in the on-line supplement, and an example of the application of the quality criteria is presented in Table 2**.**

**Table 2:** An example of the application of quality criteria

Selected study: Shirani A, Zhao Y, Karim ME, et al. Association between use of interferon beta and progression of disability in patients with relapsing-remitting multiple sclerosis. JAMA 2012;308(3):247–256. Summary This observational study investigated the association between interferon beta exposure and disability progression in patients with relapsing-remitting multiple sclerosis (MS). It was a retrospective cohort study based on prospectively collected data (1985–2008) within the British Columbia Multiple Sclerosis (BCMS) database, Canada which compared 868 patients receiving interferon with 829 untreated contemporary and 959 historical patients. The main outcome measure was time from interferon treatment eligibility (baseline) to a confirmed and sustained score of 6 (requiring a cane to walk 100 m; confirmed at > 150 days with no measurable improvement) on the Expanded Disability Status Scale (EDSS) (range, 0–10, with higher scores indicating higher disability). A multivariable Cox regression model with interferon treatment included as a time-varying covariate was used to assess the hazard of disease progression associated with interferon treatment. Analyses also included propensity score adjustment to address confounding by indication. Median active follow-up times (first to last EDSS measurement) were as follows: for the interferon–treated cohort, 5.1 years (interquartile range [IQR], 3.0–7.0 years); for the contemporary control cohort, 4.0 years (IQR, 2.1–6.4 years); and for the historical control cohort, 10.8 years (IQR, 6.3–14.7 years). The observed outcome rates for reaching a sustained EDSS score of 6 were 10.8, 5.3, and 23.1% in the 3 cohorts, respectively. After adjustment for potential baseline confounders (sex, age, disease duration, and EDSS score), exposure to interferon was not associated with a statistically significant difference in the hazard of reaching an EDSS score of 6 when either the contemporary control cohort (hazard ratio, 1.30; 95% CI, 0.92–1.83) or the historical control cohort (hazard ratio, 0.77; 95% CI, 0.58–1.02) were considered. Further adjustment for comorbidities and socioeconomic status, and separate propensity score adjustment did not substantially change the results.	
**Justification for observational design** 1. RCTs non-existent or inadequate - **Not satisfied**: A multicenter, randomized, double-blind, placebo-controlled trial of interferon beta-1b (IFNB) in 372 ambulatory patients with relapsing-remitting multiple sclerosis, entry EDSS score of 0 to 5.5 and at least two exacerbations in the previous 2 years. Patients were randomised 1:1:1 to placebo, 1.6 million international units (MIU) of IFNB, and 8 MIU of IFNB three times a week for 2 years. Annual exacerbation rates were significantly lower in both treatment groups compared with the placebo, and more patients in the 8 MIU group were exacerbation-free at 2 years compared with placebo group (*p* = 0.007). EDSS scores changed little from baseline in both the placebo and treatment arms. The IFNB Multiple Sclerosis Study Group. Interferon beta-1b is effective in relapsing-remitting multiple sclerosis. I. Clinical results of a multicenter, randomised, double-blind, placebo-controlled trial. Neurology 1993;**43**:655–61. A multicenter, randomized, double-blind, placebo-controlled trial of interferon beta-1b (IFNB) in 560 patients with entry EDSS scores of 0–5·0 were randomised 1:1:1 to interferon 22 μg, 44 μg, or placebo three times a week for 2 years. While the mean change in EDSS (− 0.25, 95% CI − 0.50 – 0.0) just reached statistical significance (*p* = 0·05), the effect was not clinically significant as the minimal clinically important difference in the 10-point EDSS scores is − 1.0. PRISMS (Prevention of Relapses and Disability by Interferon β-1a Subcutaneously in Multiple Sclerosis) Study Group. Randomised double-blind placebo-controlled study of interferon β-1a in relapsing/remitting multiple sclerosis. Lancet 1998; 352: 1498–1504. 2. RCTs not feasible – **Partially satisfied**: The authors justified their study for the following reasons: 1) typically, drug efficacy (as established through randomized clinical trials conducted under optimal conditions) is greater than drug effectiveness (as measured in “real-world” settings); 2) patients participating in clinical trials tend to be highly selected in terms of comorbidities, motivation, cognition, and ability to adhere to medication schedules; 3) follow-up protocols are highly structured, supportive, and specialized, and the duration of therapy in clinical trials is typically shorter than under usual care conditions. For all these reasons, the relationship between interferon exposure and disease progression is difficult to delineate based on clinical trials. We argue that large scale pragmatic trials with long term follow-up are possible and indeed the two RCTs cited above constituted multi-centre trials with broad inclusion criteria and follow-up of at least 2 years.	
**Minimisation of bias in study design and data collection** 1. Pre-specified study protocol: **Fully satisfied -** detailed protocol with hypothesis, participant selection criteria, data sources and collection methods, description of interventions, primary and secondary outcomes, methods of analysis and adjustment. 2. Clearly stated patient selection criteria: **Fully satisfied** – patients with confirmed diagnosis of remitting and relapsing multiple sclerosis registered with British Columbia multiple sclerosis (BCMS) clinics. Patients were either receiving interferon or not. 3. Representative study population: **Fully satisfied** – The BCMS database was established in 1980 and is estimated to capture 80% of the BC multiple sclerosis population. 4. Prospective and verifiable data collection: **Partially satisfied** – Neurologists collect clinical data relating to disease severity using standardised scoring instruments for level of disability. No mention of inter-rater reliability checks. 5. Validation checks for coded administrative data: **Not satisfied** – Data relating to exposure to interferon was collected from province-wide health administrative databases, data related to comorbid conditions captured from hospital discharge abstracts and services provided by practitioners derived from Medical Service Plan Payment Information database. No comment on validation or audit processes for ensuring accuracy and completeness of these data sources. 6. Validation checks for longitudinal data linkage processes: **Fully satisfied** – Linkage was performed through Population Data BC, a pan-provincial population health data resource, with the linkage algorithms posted on its website. Patients were identified through BCMS database and linked via their personal health number, a unique lifelong identifier. 7. Minimisation of recording bias in administrative data: **Fully satisfied** – No evidence of recording bias with no system of care incentives to upcode data to maximise revenue. 8. Minimisation of surveillance bias in clinical registry data: **Fully satisfied** – rate of clinic visits and recording of measures of disease severity disease were not different between treatment and no treatment cohorts. 9. Minimisation of recall bias**: Not applicable** 10. Minimisation of social desirability bias: **Not applicable** 11. Independent assessment of outcomes: **Not satisfied** – Clinicians caring for patients were the same individuals scoring measures of disease severity and who were aware of whether patient was receiving interferon or not. No comment on measures being performed, even in a random subset, by independent assessors blind to treatment status.	
**Appropriate methods used to create comparable groups** 12. Appropriate statistical regression models for balancing populations: **Fully satisfied** – A multivariable Cox regression model with interferon beta treatment included as a time-varying covariate was used to assess the hazard of disease progression with treatment. Analyses also included propensity score adjustment to address confounding by indication. 13. Model includes all important predictor variables: **Partially satisfied** – Model was adjusted for sex, age, disease duration, EDSS score at baseline, socioeconomic status and Charlson co-morbidity index. However other potentially relevant predictors were absent: previous relapse rate, prior use of other treatments such as high dose steroids and copaxone, clinic volumes (as proxy measures of clinician experience) 14. Appropriate selection and measurement of all important predictor variables: **Fully satisfied –** Selection of variables is self-explanatory and detailed discussion of how they were measured. 15. Majority of population sample included in analysis: **Fully satisfied** – more than 80% of the sample was included. 16. Imputation methods for missing data: **Not satisfied -** patients with fewer than 2 prospective EDSS measurements from baseline to study end were excluded; however, patients with more than 2 measurements but then lost to follow-up were censored at the last recorded EDSS measurement, and this measurement included in the Cox proportional hazards model. No comment as to what proportion of the sample comprised patients lost to follow-up. 17. Comparison groups well balanced: **Not applicable** – used Cox multivariable regression models for primary analysis	
**Appropriate adjustment of observed effects** 18. Subgroup analyses and Interaction testing for identifying independent prognostic variables: **Not satisfied** – no comments made as to interaction testing or subgroup analyses 19. Avoidance of unplanned post-hoc analysis: **Fully satisfied** – no post-hoc analyses reported 20. Correction for multiple outcome analyses: **Not applicable** – there was only one outcome measure: change in EDSS over time. 21. Adjustment for clustering effects in multicentre studies: **Not satisfied** – there were 4 separate clinics so clustering effects could operate, especially as the clinicians in each who were reporting EDSS were not blind to treatment and there may be inter-clinic differences in other co-interventions 22. Adjustment for time-dependent bias: **Fully satisfied** – disease duration and timing of interferon treatment were treated as time-varying co-variates in Cox proportional hazards model.	
**Validation of observed effects** 23. Large effect size: **Not applicable** – there was no difference between groups 24. Exclusion of possible benefit in presence of negative results: **Not satisfied** – the point estimate of the hazard ratio comparing interferon treated with contemporary untreated patients was 1.30 with the upper 95% CI limit of 1.83, suggesting potential for benefit despite non-significant result with lower 95%CI limit being 0.92. 25. Sensitivity analysis for unmeasured confounders: **Not satisfied** – no analyses done 26. Plausibility of intervention mechanism of action: **Fully satisfied** – biologically plausible 27. Temporal relation between intervention and outcomes: **Fully satisfied** – extended period of follow-up for all three cohorts which gave adequate time for benefit to show if treatment was effective 28. Dose-response relationship: **Not applicable** – as no effect was seen 29. Consistency with other studies of same intervention: **Fully satisfied** – previously performed randomised trials and systematic review have not confirmed that interferon has clinically meaningful effects on the disease course 30. Coherence with other studies of similar interventions: **Not applicable** – no other similar interventions at the time – in later years a pegylated form of interferon was entered into trials 31. Falsification test for intervention effect specificity: **Not satisfied** – no mention of falsification tests in the study protocol in the event of benefit or harm being observed.	
**Authors interpretations** 32. Study limitations acknowledged – **Fully satisfied** – extensive discussion stating limitations such as differential patient recruitment according to disease severity, limited choice of disease outcome measures and limitations of EDSS to assess all relevant functional domains, and inadequate study power. 33. Impartial statement of study implications: **Partially satisfied** – authors state their findings question the routine use of interferon in preventing or delaying long-term disability, despite previously stating the limitations of the EDSS. They do concede that it is possible a subgroup of patients may benefit, and may be identified using pharmacogenomic or biomarker studies.	

### Analyses of methodological quality and risk of bias

**Fig. 1:**
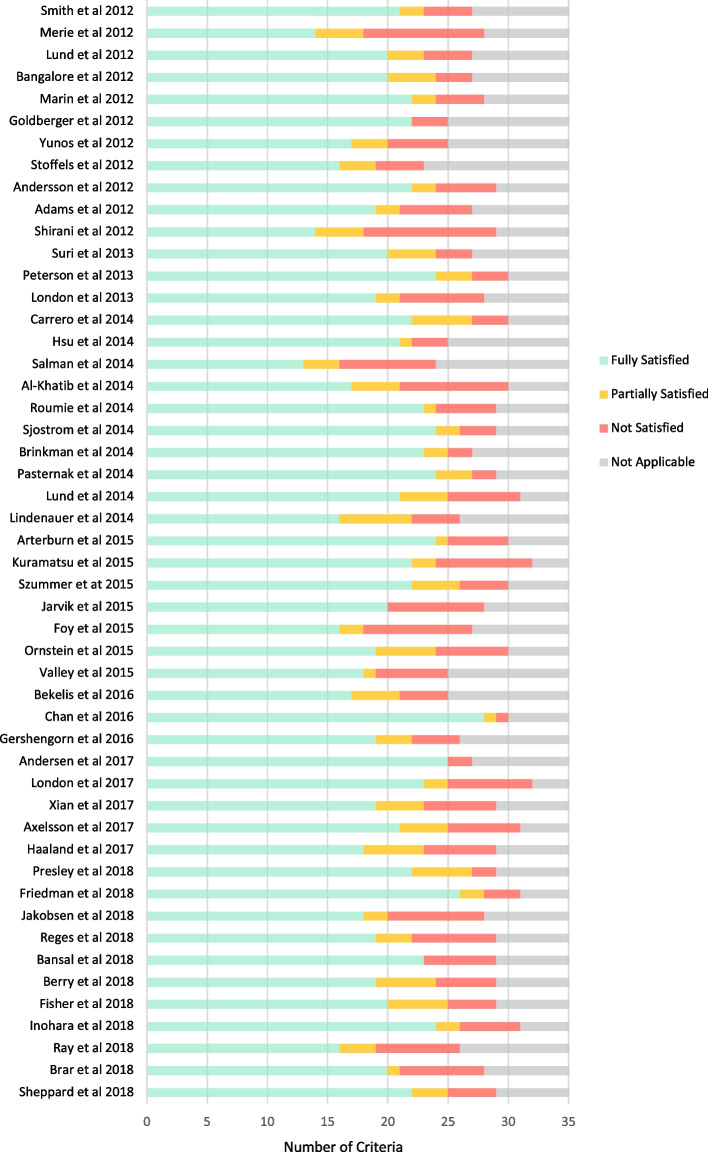
Concordance with all quality criteria for individual studies

The proportions of applicable criteria which were fully, partially or not satisfied for each study are depicted in Fig. 1. No study was shown to have all applicable criteria fully satisfied, with the mean (+/−SD) proportion of applicable criteria fully satisfied across all studies being 72% (+/− 10%). This figure was the same for both non-procedural (68% [+/− 9%]) and procedural (70% [+/− 10%]) interventions. The categories of quality criteria demonstrating the lowest proportions of fully satisfied criteria were measures used to adjust observed effects (criteria 20, 23, 24) and validate observed effects (criteria 25, 27, 33).

At the level of individual studies, the proportions of all criteria fully or partially satisfied ranged between 60.7% (17 of 28 criteria) and 96.6% (28 of 29 criteria) and the proportions of all criteria that were not satisfied ranged from 3.4% (1 of 29 criteria) to 39.3% (11 of 28 criteria). Only two studies had more than 80% of applicable criteria fully satisfied (Chan et al. at 87% [50] and Friedman et al. at 81% [58];) while two studies met only 50% of applicable criteria (Merie et al. [19]; Shirani et al [28]).

**Fig. 2:**
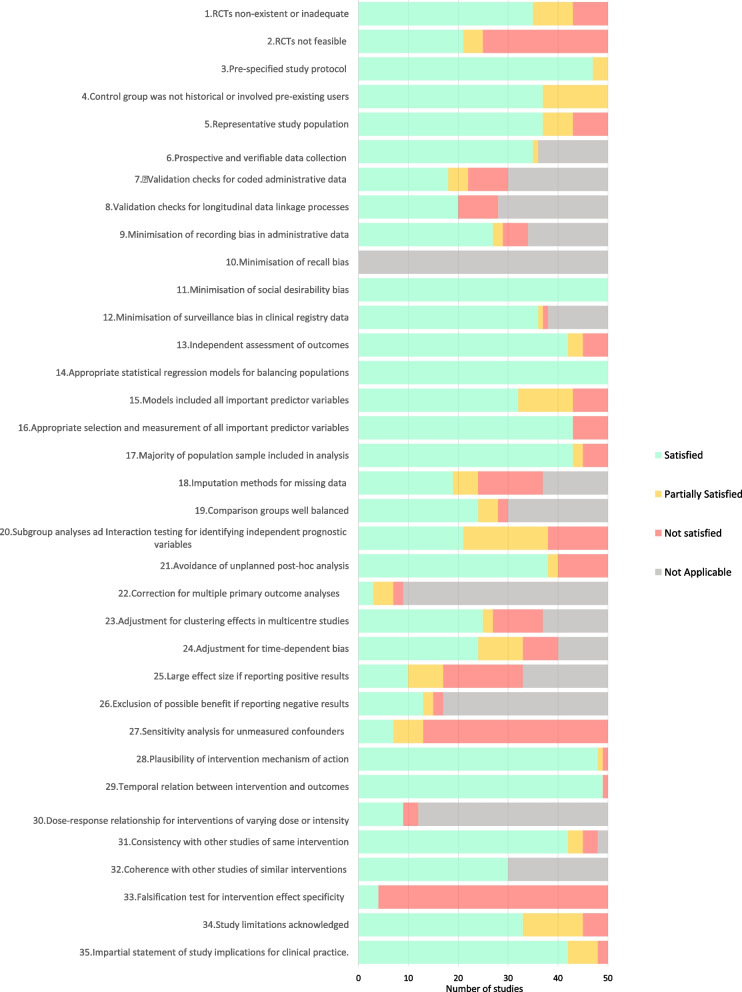
Concordance with individual quality criteria for all studies combined

At the level of individual criteria, the proportions of studies in which a specific criterion was fully, partially and not satisfied, or was not applicable, are depicted in Fig. 2**.**  One criterion (recall bias) was not applicable to any study as informal patient self-report was not used as a data source.

Across all studies, criteria associated with high levels (≥80%) of full satisfaction (where applicable) comprised: appropriate statistical methods (most commonly propensity-based methods) used to ensure balanced groups (100%); absence of social desirability bias as all studies either used validated, externally administered questionnaires or did not rely on patient self-reported symptoms or function as their primary end-points (100%); coherence of results with other studies of similar interventions (100%); temporal cause-effect relationships (98%); prospective, validated data collection (97%); plausibility of results (96%); absence of surveillance bias in clinical registry data (95%); formulation of pre-specified study protocol (94%); consistency of results to similar studies of same interventions (88%); clear statements on how prognostic variables were selected and measured (86%); data from the majority of the population sample being used in analyses (86%); absence of overstatement of study conclusions (84%); independent blind assessment of outcomes (84%); and adequate matching of patient populations being compared (80%).

Criteria associated with intermediate (51 to 79%) levels of full satisfaction comprised: absence of recording bias in administrative datasets (79%); presence of dose-response relationships (75%); absence of unplanned post-hoc analyses (76%); statistical exclusion of potentially beneficial effect in studies with conclusions of no effect or harm (76%); adequate accounting for selection bias in patient recruitment (74%); and representativeness of the study population (74%).

Criteria associated with low (≤50%) levels of full satisfaction comprised: imputation or other processes to account for missing data or drop-outs (50%); justification for not performing an RCT (42%); interaction analyses in identifying independent prognostic factors that may have influenced intervention effects (42%); use of statistical correction methods to minimise type 1 error arising from multiple analyses of several different outcome measures (33%); clinically significant effect sizes (30%); residual bias analyses that accounted for unmeasured or unknown confounders (14%); and falsification tests for residual confounding (8%).

### Trend analysis

**Fig. 3:**
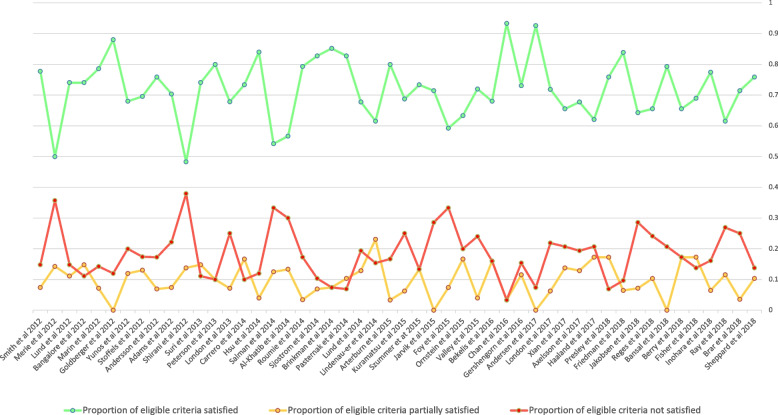
Trend analysis of quality criteria concordance over time

The proportions of all applicable criteria that were fully, partially or not satisfied showed no appreciable change over time (Fig. 3).

## Discussion

To our knowledge, this is one of only a few studies to apply a comprehensive list of criteria for assessing the methodological rigour of a cohort of contemporary observational studies of commonly used therapeutic interventions in adult patients reported in high-impact general medicine journals. Overall, there was a high level of adherence to criteria related to study protocol pre-specification, sufficiently sized and representative population samples, prospective collection of validated and objective data with minimisation of various forms of ascertainment and measurement bias, appropriate statistical methods, avoidance of post-hoc analyses, testing for causality, and impartial interpretation of overall study results. These criteria are central to most critical appraisal guides and reporting guidelines for observational studies, are well known to researchers, and hence will likely attract a high level of adherence.

However, there is room for improvement. On average, each study failed to satisfy at least one in four quality criteria which were applicable to that study. The most frequent omission was failing to conduct a falsification (or specificity of effect) test for studies which reported intervention benefits. This test demonstrates whether a benefit is seen for outcomes that can be plausibly attributed to the intervention (eg reduction in myocardial infarctions with coronary revascularisation), but no change for a separate outcome most unlikely to be affected by the intervention (eg in this example, reduction in cancer incidence), whereas if a benefit is seen for both outcomes, then the intervention is probably not the causative factor but some other confounding factor that affects both outcomes [68]. Second was the failure to eliminate the possibility of positive effects being annulled or attenuated by an unmeasured or unknown confounder by undertaking residual (or quantitative) bias or instrumental variable analyses. A new concept called the ‘E value’ and its associated formula have recently been articulated which denotes how prevalent and sizeable in its effects such a confounder would have to be to negate the observed benefit [69, 70]. Understandably, as this is a recent innovation, studies prior to 2017 could not have used this technique, although other methods have been used in the past [71], and this form of bias has been known for decades [72]. Third was the absence of large effect sizes which, according to GRADE, lessens the likelihood that the observed benefit is real, as small effect sizes provide little buffer against residual confounding [73]. Exactly what constitutes a large enough effect size to counter such confounding remains controversial, with relative risks (RRs) > 2 (or < 0.5) [74], ≥5 (or ≤ 0.2) [73] or ≥ 10 (or ≤ 0.1) [75] being cited as reasonable thresholds. We chose the first of these three thresholds as the minimum necessary, cognizant of the fact that RRs varying between 0.5 and 2 are the ones most commonly reported. Fourth was the absence of correction for statistical significance (using Bonferroni or other methods) for multiple outcome analyses in avoiding type 1 errors whereby significant but spurious benefits are generated simply by the play of chance [76]. Fifth was the omission of subgroup analyses and statistical interaction testing that could identify effect modifiers that differentially influence intervention effects [77]. Proper use of such analyses seems to be an ongoing challenge for RCTs as well [78]. Sixth was lack of multiple imputation processes to account for missing data or drop-outs, an omission frequently seen in clinical research [79]. Such analyses assess the potential for observed effects to have been attenuated by unascertained adverse events occurring among those lost to follow-up at study end, particularly if the outcome of interest, such as deaths, is infrequent. Finally, many studies failed to provide a substantive reason why an RCT could not be performed in the absence of existing RCTs. While it may arguably not qualify as a quality criterion, we believe researchers are obliged to explain why a study design vulnerable to bias was preferred over more robust randomised designs if no substantive barriers to doing such an RCT existed.

A further concern is that despite the promulgation of reporting guidelines for non-randomised studies and the development of statistical methods for gauging the level of sensitivity of results to residual bias, our trend analysis indicates little improvement in methodological quality of studies published between 2012 and 2018. Overall, deficits in statistical analytic methods featured more prominently than deficits in study design and conduct. In particular, the absence of falsification tests, E-value quantification, subgroup analyses using tests for interaction, and adjustment for missing data and multiple comparisons limited the ability of many studies to account for residual confounding in their results.

### Limitations

Our study has several limitations. First, despite excellent agreement between authors in categorising levels of criterion satisfaction, this task involves subjective and potentially biased judgement. However, this problem is common to most quality assessment tools [80]. Second, our criteria have not been validated, although few tools have, and, in any event, our criteria included those contained within other well-publicised instruments which have recognised limitations [81]. Third, some may argue that studies not using propensity score methods to create matched cohorts for primary analyses and relying solely on multivariate regression models should be classed as more vulnerable to bias than those which do. However, research has not shown the former to be necessarily superior to the latter [82]. Fourth, we made no attempt to rank or weight criteria according to the magnitude of their potential to bias study results, but as far as we aware, no validated weighting method has been reported [83]. Fifth, our chosen threshold for effect size (odds ratio ≤ 0.5 or relative risk reduction ≥50%) is arbitrary and may be regarded as too stringent, but is the upper threshold quoted by other researchers [73–75]. Sixth, our small sample of 50 studies, with the majority taken from only 2 journals, and identified from searching only one database is arguably not representative of all observational studies of therapeutic interventions, although PubMed is the database widely used by practising clinicians to find articles most relevant to their practice. The inclusion of the terms ‘association’ and ‘observational’ in our search strategy likely biased study retrieval towards articles published in JAMA and JAMA Internal Medicine, as these journals use these words consistently in their titles and abstracts. However, it is also possible these journals have a greater propensity than other journals to publish observational studies. We would recommend that all journals request authors to have their study titles clearly indicate they are observational. While the sample is small, the included studies involved commonly used clinical interventions, and by being published in high impact journals have considerable potential to influence practice. Moreover, other investigators have found it difficult to find large numbers of observational trials in specific disciplines over extended periods of time [84].

## Conclusion

Contemporary observational studies published in two high impact journals show limitations that warrant remedial attention from researchers, journal editors and peer reviewers. Reporting guidelines for such studies should promulgate the need for falsification testing, quantification of E values, effects sizes that denote less vulnerability to residual confounding, appropriate statistical adjustment for multiple outcome analyses, statistical interaction tests for identifying important predictors of intervention effects, complete patient follow-up, and justification for choosing to undertake an observational study rather than an RCT.

## Supplementary Information

**Additional file 1.** Study characteristics.

## Data Availability

All data generated or analysed during this study are included in the manuscript and in the appendix.
