# Supplementary material for: Quality of observational studies of clinical interventions: a meta-epidemiological review
Source: BMC Med Res Methodol. 2022 Dec 7;22:313. doi: 10.1186/s12874-022-01797-1 (PMC9727931; doi:10.1186/s12874-022-01797-1)
Supplement: Supplementary file 1 — Additional file 1. Study characteristics. [file 12874_2022_1797_MOESM1_ESM.docx]

**On-line supplement. Study characteristics**

| **Authors, study date (reference)** | **Comparator interventions** | **Study population and data source** | **Statistical methods used to balance and/or analyse groups** | **Outcome measures** | **Principal findings** |
| --- | --- | --- | --- | --- | --- |
| Smith et al 2012 (18) | Brachytherapy vs whole breast irradiation (WBI) in older women with breast cancer. | Retrospective population-based cohort study of women aged 67 years or older with incident invasive breast cancer with fee-for-service Medicare, diagnosed between 2003 and 2007 and followed up through 2008. Subset of lumpectomy patients treated with brachytherapy (n=6952) vs WBI (n=85,783). | Multivariate proportional hazards models tested association between radiation treatment (brachytherapy vs WBI) and subsequent mastectomy and adjusted for covariates identified a priori based on clinical significance and univariate analyses. | Cumulative incidence and adjusted risk of subsequent mastectomy (an indicator of failure to preserve the breast) and death. | Treatment with brachytherapy compared with WBI was associated with worse long-term breast preservation and increased complications but no difference in survival. |
| Merie et al 2012 (19) | Warfarin versus no warfarin following aortic valve replacement (AVR) with biological prostheses. | Retrospective cohort study of patients in Danish National Patient Registry who had bioprosthetic AVR between 1/1/1997 and 31/12/2009. Concomitant comorbidity and medication were retrieved (n=4,075). | Poisson regression models were used for survival analysis to calculate incidence rate ratios as an indicator of relative risk (RR). | Incidence rate ratios (IRRs) of strokes, thromboembolic events, cardiovascular deaths, and bleeding incidents by discontinuing warfarin vs continued treatment 30 to 89 days, 90 to 179 days, 180 to 364 days, 365 to 729 days, and at least 730 days after surgery. | Discontinuation of warfarin treatment within 6 months after bioprosthetic AVR surgery was associated with increased cardiovascular death. |
| Lund et al 2012 (20) | Renin angiotensin system (RAS) antagonists versus no RAS antagonists in patients with heart failure with preserved ejection fraction (HFpEF). | Prospective study using the Swedish Heart Failure  Registry of unique patients registered from 64 hospitals and 84 outpatient clinics between 2000 and 2011. (n=16,216). | Cohort was matched 1:1 based on age and propensity score and in the overall cohort with adjustment for propensity score as a continuous covariate. Separate age and propensity score–matched analyses were performed according to RAS antagonist dose. | All-cause mortality at 12 months. | In the matched cohort, 1-year survival was reduced by 9% in patients receiving RAS antagonists, which increased to 15% reduction in patients receiving 50% or greater of target dose. |
| Bangalore et al 2012 (21) | β-blocker vs no β-blocker in stable patients with prior history of myocardial infarction (MI), in patients with coronary artery disease (CAD) but no history of MI, and in patients with only risk factors for CAD. | Retrospective cohort study of patients in Reduction of Atherothrombosis for Continued Health (REACH) registry divided into 3 cohorts: known prior MI (n=14 043), known CAD without MI (n=12 012), no CAD with risk factors only (n=18,653). | Propensity score matching was performed using 27 baseline variables. Regression adjustment with propensity score (as a continuous variable) was also performed using all patients in the cohort. | Composite of cardiovascular death, nonfatal MI, or nonfatal stroke. Secondary outcome of primary outcome plus hospitalization for atherothrombotic events or revascularization procedure at 4 years. | β-blockers were not associated with lower risk of composite cardiovascular events among patients with either CAD risk factors only, known prior MI, or known CAD without MI. |
| Marin et al 2012 (22) | Continuous positive airway pressure (CPAP) therapy vs no CPAP in patients with obstructive sleep apnoea (OSA). | Retrospective cohort study of participants without hypertension referred to a sleep center in Zaragoza, Spain, for nocturnal polysomnography between 1994 and 2000 (n=1889). | Multivariable models adjusted for confounding factors, including change in body mass index from baseline to censored time, were used to calculate hazard ratios (HRs). | Incidence of new-onset hypertension over 12 months. | Compared with controls, incident hypertension was 33% greater among OSA patients ineligible for CPAP therapy, 96% greater among those who declined CPAP therapy, and 78% greater among those nonadherent to CPAP therapy; but 29% lower in patients treated with CPAP therapy. |
| ldberger et al 2012 (23) | Longer vs shorter duration of cardiopulmonary resuscitation for in-hospital cardiac arrest. | Retrospective cohort study of patients with cardiac arrests at 435 US hospitals enrolled in the Get With The Guidelines—Resuscitation registry between 2000 and 2008 (n=64 339). | Multilevel regression models were used to assess association between the length of resuscitation attempts and risk-adjusted survival. | Immediate survival with return of spontaneous circulation during cardiac arrest and survival to hospital discharge. | Compared with patients at hospitals in the quartile with shortest median resuscitation attempts in non-survivors (16 min), those at hospitals in quartile with longest attempts (25 min) had a risk-adjusted increase of 12% in return of spontaneous circulation and survival to discharge. |
| Yunos et al 2012 (24) | Chloride-restrictive vs chloride-liberal intravenous fluid strategy with acute kidney injury (AKI) in critically ill patients. | Prospective open-label, sequential period pilot study of consecutive patients admitted to intensive care unit (ICU) of tertiary hospital in Melbourne, Australia during control period (18/2/2008-17/8/2008; n=760) compared with consecutive patients admitted during intervention period (18/2/2009-17/8/2009; n=773). | Generalized linear modelling was used for continuous variables; logistic regression models and time-to-event analyses using Cox proportional hazard modelling were used for defined events. | Increase from baseline to peak creatinine level in the ICU; incidence of AKI according to the risk, injury, failure, loss, end-stage (RIFLE) classification; incidence of renal replacement therapy (RRT). | Adjusted analyses showed chloride-restrictive strategy was associated with reduced rise in serum creatinine and 48% decrease in odds of incidence of injury and RIFLE-defined AKI and use of RRT. |
| Stoffels et al 2012 (25) | Single-photon emission computed tomography/computed tomography (SPECT/CT)-aided sentinel lymph node excision (SLNE) vs standard SLNE in patients with melanoma. | Retrospective analysis of computerized melanoma patient database at University Hospital Essen, Skin Cancer Center, Essen, Germany, used to identify patients eligible for SLNE between March 2003 and April 2011 (n=464). Subset of 403 patients with clinically negative lymph nodes who underwent SLNE with or without preoperative SPECT/CT. | Univariate analysis and stepwise multivariate Cox proportional  hazards regression model were used to analyse association between SPECT/CT and metastatic node detection and survival adjusted for histology, sex, localization of the primary, age, Breslow thickness, ulceration, and SLN status as prognostic factors. | Metastatic node detection and disease-free survival at 4 years. | SPECT/CT allowed SLNE in the head and neck area to occur more frequently (23.5% vs 2.0%); it detected more sentinel lymph nodes per patient (2.40 vs 1.87); and was associated with lower local relapse rate (6.8% vs 23.8%) and longer 4-year disease-free survival (93.9% vs 79.2%). |
| Andersson et al 2012 (26) | Clopidogrel treatment after myocardial infarction (MI) in patients with diabetes vs similar patients with no diabetes. | Retrospective cohort study of individual-level linked nationwide Danish  administrative registries between 2002-2009 of patients hospitalized with incident MI and who survived without undergoing coronary artery bypass surgery, and followed up for as long as 1 year (n= 58,851). | Cox proportional-hazard models  and propensity score−matched models were used, adjusted for age, sex, comorbidity, calendar year, concomitant pharmacotherapy,  and invasive interventions. | All-cause mortality, cardiovascular mortality, and composite  end point of recurrent MI and all-cause mortality at 12 months. | Clopidogrel was associated with less effectiveness in diabetic vs non-diabetic patients for all-cause mortality (no difference vs 25% reduction in non-diabetics) and for cardiovascular mortality (no difference vs 23% reduction). No difference was seen between groups for composite end point. |
| Adams et al 2012 (27) | Roux-en-Y gastric bypass (RYGB) surgery vs nonsurgical management in morbidly obese patients. | Retrospective cohort analysis of adult patients entered into Utah-based single-centre registry study between 2000 and 2011 who were severely obese (body mass index [BMI] ≥35) and who sought and received RYGB surgery (n=418), sought but did not have surgery (n=417; control group 1), or were randomly selected from a population-based sample not seeking weight loss surgery (n=321; control group 2). | Propensity scores were created from logistic regression model regressing baseline group membership on baseline values of sex, age, body mass index, income, education level, and marital status. Propensity scores were adjusted for baseline variable distribution differences  among study group. Logistic regression used to analyse group differences in incidence  and remission of disease end  points (diabetes, dyslipidemia, hypertension). | Weight loss, diabetes, hypertension, dyslipidemia, and health-related quality of life at 6 years. | Use of RYGB surgery was associated with higher rates of diabetes remission (62% vs 8%/6%); hypertension remission (45% vs 18%/9%). |
| Shirani et al 2012 (28) | Interferon beta vs no interferon beta therapy in patients with relapsing-remitting multiple sclerosis (MS). | Retrospective cohort study of patients entered into British Columbia Multiple Sclerosis (BCMS) database linked with province-wide health administrative databases including PharmaNet for exposure to interferon beta; Medical Service Plan Payment Information, and Discharge Abstracts for hospital separations and pre-existing comorbid conditions; and Census Geodata for socioeconomic status (SES) data (interferon beta [n=868]; untreated contemporary  [n=829] and historical [n=959] cohorts). | Multivariable Cox regression model was used with interferon beta treatment included as a time-varying covariate to assess hazard of disease progression associated with interferon beta treatment. Analyses also included propensity  score adjustment. | Time from interferon  beta treatment eligibility (baseline) to a confirmed and sustained score of 6 (requiring a cane to walk 100 m; confirmed at >150 days with no measurable improvement) on  the Expanded Disability Status Scale (EDSS) (range, 0-10, with higher scores indicating higher disability). | Administration of interferon beta was not associated with reduction in progression of disability. |
| Suri et al 2013 (29) | Nonsurgical observation vs early mitral valve surgery following the diagnosis of mitral  regurgitation due to flail leaflets. | Retrospective study of consecutive patients with flail mitral valve regurgitation in Mitral Regurgitation International Database (MIDA) registry (1980-2004)  and receiving routine cardiac care from 6 tertiary centers (France, Italy, Belgium, and the United States) (n= 2097). | Cox proportional hazards regression, propensity score matched cohorts and inverse probability weighed (IPW) analyses were used. | All-cause survival and incidence of heart  failure and new-onset atrial fibrillation over 10 years. | Long-term survival rates were higher for patients with early surgery (86% vs 69% at  10 years), confirmed in adjusted models (hazard ratio [HR] 0.55), propensity-matched cohort (HR 0.52) and IPW analysis (HR, 0.66). Long-term heart failure risk was also lower (7% vs 23%) confirmed in risk-adjusted models (HR 0.29), propensity-matched cohort (HR, 0.44) and in IPW analysis (HR, 0.51). No reduction was seen in late-onset atrial fibrillation. |
| Peterson et al 2013 (30) | Single vs dual-chamber implantable cardioverter-defibrillators (ICDs) for primary prevention  of sudden cardiac death. | Retrospective cohort study of admissions in the National Cardiovascular Data Registry’s (NCDR) ICD registry from 2006-2009 linked to fee-for-service Medicare claims data (n=32,034). | Propensity score matched cohorts with matched McNemar tests were performed to determine whether rates of subsequent complications, mortality, all-cause admission, and heart failure admission differed between groups. Multivariable Cox proportional survival models to account for clustering among hospitals. | Adjusted risks of 1-year mortality, all-cause readmission,  heart failure readmission, and device-related complications within 90 days. | Single-chamber device was associated with lower risk of device-related complications (3.5% vs 4.7%) and similar 1-year mortality and hospitalization rates. |
| London et al 2013 (31) | Early perioperative use (day before or shortly following) of β-blockers vs no β-blockers in patients undergoing noncardiac surgery. | Population-based cohort analysis of patients treated at 104 Veterans Affairs medical centers from January 2005 to August 2010. (n=136,745). | 1:1 propensity-score matched cohorts were used with stratified analyses according to scores on Revised Cardiac Risk Index (RCRI). | All cause 30-day mortality and cardiac morbidity  (cardiac arrest or Q-wave myocardial infarction). | β-blocker use was associated with overall 27% lower mortality. When stratified by RCRI, β-blocker use was associated with 37%, 46% and 60% lower mortality among patients with 2, 3 or ≥4 factors undergoing nonvascular surgery. β-blocker use was associated with 33% lower rate of nonfatal Q-wave infarction or cardiac arrest in patients undergoing nonvascular surgery. |
| Carrero et al 2014 (32) | Warfarin vs no warfarin patients with recent myocardial infarction (MI) and atrial fibrillation (AF) stratified according to severity of co-existent chronic kidney disease (CKD). | Multicenter cohort study from the Swedish Web-System for Enhancement and Development of Evidence-Based Care in Heart Disease Evaluated According to Recommended Therapies (SWEDEHEART) registry  (2003-2010), which includes all Swedish hospitals that provide care for acute cardiac  Diseases.Consecutive survivors of an acute MI with AF and known serum creatinine and estimated glomerular filtration rate (eGFR) (n = 24,317). | Two sets of multivariable  Models were used:  (1) adjusted for age, sex, eGFR, pre-existing comorbidities,  patient presentation characteristics at admission, hospital course, discharge medication and center effect and frailty.  (2) further adjusted for left ventricular ejection fraction.  Two sensitivity analyses were performed using complete-case analysis and propensity scores within a Cox regression with warfarin at discharge and frailty as predictors. | (1) Composite end point of death, readmission due  to MI, or ischemic stroke; (2) bleeding (composite of readmission due to hemorrhagic stroke,  gastrointestinal bleeding, bleeding causing anemia, and others); or (3) aggregate of these 2 outcomes within 1 year from discharge date. | Warfarin treatment was associated with on average 24% lower 1-year risk for composite outcome of death, MI, and ischemic stroke without a higher risk of bleeding. This association was not related to the severity of concurrent CKD. |
| Hsu et al 2014 (33) | Renin-angiotensin-aldosterone system (RAS) blocker vs no RAS blockers in patients with advanced (pre-dialysis) chronic kidney disease (CKD) (n=28,497). | Population-based cohort study of patients identified as having advanced CKD from the National Health Insurance Research Database in Taiwan on the basis of ICD-9 codes and with data on date of birth, sex, residency area, diagnostic codes, drug prescriptions, and medical procedures. Users (n = 14,117) and nonusers (n = 14,380) of RAS blockers. | Multivariate Cox proportional hazards regression models were used, adjusted for age, sex, Charlson comorbidity index, diabetes mellitus, coronary artery disease, stroke, cancer, frequency of visits to nephrologists within 3 years before the index date, geographic location, and non-RAS antihypertensives. Sensitivity analyses included propensity-score matched pairs. | Commencement of long-term dialysis and all-cause mortality at 7 months | Use of RAS blockers ssociated with 6% lower risk for long-term dialysis and for composite of long-term dialysis or death. Compared with nonusers, RAS blockers had a higher risk of hyperkalemia-associated hospitalization (9.2% vs 6.7%) but all-cause mortality was not affected. |
| Salman et al 2014 (34) | Conservative management vs interventional treatment for unruptured brain arteriovenous malformations (bAVMs). | Population-based cohort study using anonymized data extracted from the National Health Service Scottish Audit of Intracranial Vascular Malformations comprising Scottish residents aged 16 years or older when first  diagnosed as having a bAVM in 1999-2003 or 2006-2010 (n=136,745). | Multivariate Cox proportional hazards regression model was used adjusted for age at inception, mode of clinical  presentation, baseline occurrence of handicap scores (for primary outcome only), and vascular anatomy that influenced the risk of bAVM hemorrhage according to bAVM nidus location, bAVM venous drainage pattern or risk of intervention, and maximum bAVM nidus diameter. Sensitivity analyses were performed using adjusted Cox models according to propensity scores. | Rates of primary outcome (death or sustained morbidity of any cause by Oxford Handicap Scale  [OHS] score ≥2 for≥2 successive years [0 = no symptoms and 6 = death]) and the secondary outcome (nonfatal symptomatic stroke or death due to bAVM, associated arterial  aneurysm, or intervention) at 12 years. | Rate of progression to primary outcome was 41% lower with conservative  management during the first 4 years of follow-up (9.5 vs 9.8 per 100  person-years), but rates were similar  thereafter. Rate of secondary outcome was 63% lower with conservative management during 12 years of follow-up (1.6 vs 3.3 per 100 person-years). |
| Al-Khatib et al 2014 (35) | Prophylactic implantable  cardioverter-defibrillators (ICDs) vs no ICD in patients with left ventricular ejection fraction (LVEF) 30% to 35%. | Population-based cohort study of Medicare beneficiaries in the National Cardiovascular Data Registry ICD registry (1/1/2006 to 31/12/2007) with an LVEF 30%-35% who received an ICD during a heart failure  hospitalization and similar patients in the Get With The Guidelines–Heart Failure (GWTG-HF) database (1/1/2005 to 31/12/2009) with no ICD (n=7,698). | Propensity score matched cohorts with Cox proportional hazards models were applied to matched cohorts to map ICD with mortality. | All-cause mortality at 3 years. | ICD was associated with 17% reduction in all-cause mortality in patients (51.4% vs 55.0%). |
| Roumie et al 2014 (36) | Insulin vs sulphonylureas in diabetic patients receiving metformin. | Population-based cohort study of US veterans initially treated with metformin from 2001 through 2008, with data derived from National  Veterans Health Administration, Medicare, and National Death Index databases (n=178,341). | Propensity score matching was performed of one participant who added insulin to 5 who added sulfonylurea.  Comparisons were undertaken using marginal structural Cox  proportional hazard models adjusted for baseline and time-varying demographics,  medications, cholesterol level, hemoglobin A1c level, creatinine level, blood pressure, body  mass index, and comorbidities. | Composite outcome of acute myocardial infarction (AMI), stroke hospitalization,  or all-cause death at 14 months. | Insulin was associated with 30% increased risk of composite outcome compared to sulphonylurea (42.7 vs 32.8 events per 1000 person-years) and 44% increase in risk of all-cause death (33.7  and 22.7 events per 1000 person-years). No differences were seen in rates of AMI, stroke, hospitalizations, or cardiovascular deaths. |
| Sjostrom et al 2014 (37) | Bariatric surgery vs no bariatric surgery in morbidly obese patients with type 2 diabetes. | Prospective cohort study (Swedish Obese Subjects (SOS)) conducted at 25 surgical departments and 480 primary health care  centers in Sweden (n=4,047). | Bariatric surgery patients were matched with control patients on the basis of 18 variables from a matching examination and identical inclusion and exclusion criteria. Multivariable logistic regression models were used to identify predictors for both diabetes remission and relapse. Cox proportional hazards regression models were used to analyze time to event curves. | Diabetes remission, relapse, and diabetic complications. Remission was defined as blood glucose <110 mg/dL and no diabetes medication at 2 and 15 years. | Bariatric surgery patients showed a 13-fold greater remission rate at 2 years (16.4% vs 72.3%) and 6-fold greater remission rate at 15 years (6.5% vs 30.4%) compared to no surgery. Cumulative incidence rates of microvascular complications were decreased by 56% (41.8 vs 20.6 per 1000 person-years). Macrovascular complications were reduced by 32% (44.2 vs 31.7 per 1000 person-years). |
| Brinkman et al 2014 (38) | Preoperative β-blocker use in non-emergent coronary artery bypass surgery. | Society of Thoracic  Surgeons National Adult Cardiac database for 1107 hospitals performing cardiac surgery in US from 1/1/2008 to 31/12/2012 (n=506,110). | Multiple logistic regression and propensity matching were used with a greedy 5-to-1 digit-matching algorithm. | Incidence of perioperative mortality, permanent stroke, prolonged ventilation, any reoperation, renal failure, deep sternal wound infection, and atrial  fibrillation at 30 days post-procedure. | No differences were seen between groups blockers in rates of operative mortality, stroke, prolonged ventilation, any reoperation, renal failure, deep sternal wound infection. Risk of new-onset atrial fibrillation was increased by 9% with β-blocker use. |
| Pasternak et al 2014 (39) | Carvedilol vs metoprolol succinate in patients with heart failure (LVEF <40%). | Danish national Heart Failure registry linked with health care and administrative databases (n=11,664). | Cox regression was used with adjustment for a propensity score, derived from a range of clinical, socioeconomic, and demographic characteristics. | All-cause mortality (primary outcome) and cardiovascular mortality (secondary outcome) at 2.4 years. | No differences were seen between carvedilol and metoprolol succinate in all-cause or cardiovascular mortality. |
| Lund et al 2014 (40) | β-blocker use in patients with heart failure and preserved ejection fraction (HFpEF) vs no β-blocker use. | Nationwide Swedish Heart Failure Registry of 67 hospitals and 95 primary care clinics (n=19,083). | Propensity score–matched cohort study was performed, with propensity scores derived from 52 baseline clinical and socioeconomic variables. Additional positive-control consistency analysis was performed involving 22,893 patients with heart failure with reduced ejection fraction (HFrEF)with 4054 patients treated with β-blocker and 2027 untreated patients. | Primary outcome of all-cause mortality; secondary outcome of composite of all-cause mortality or heart failure hospitalization at 1 and 5 years. | Survival at 1 year was no different but at 5-years mortality was lower in β-Blocker group (42% vs 45%; hazard ratio [HR],0.93). β-blockers were not associated with reduced combined mortality or heart failure hospitalizations.  In matched HFREF cohort, β-blockers were associated with reduced mortality (HR, 0.89) and with reduced combined mortality or heart failure  hospitalization (HR 0.89). |
| Lindenauer et al 2014 (41) | Noninvasive (NIV) vs invasive (IMV) ventilation in patients with severe exacerbations of chronic obstructive pulmonary disease (COPD). | Retrospective cohort study of  patients hospitalized for exacerbation of COPD at 420 US hospitals participating in the Premier Inpatient  Database (n=25,628). | Propensity model for treatment with NIV was used that included all patient and hospital characteristics and treatments for COPD. A series of hierarchical generalized linear  models were used to assess independent effect of NIV on study outcomes, adjusted for patient, physician, and hospital characteristics, and the propensity for treatment with NIV. | In-hospital mortality, hospital-acquired pneumonia, hospital length of stay and cost, and 30-day readmission. | NIV was associated with lower mortality risk than IMV (OR 0.54; [95%CI, 0.48-0.61]), lower risk of hospital-acquired pneumonia (OR, 0.53 [95%CI, 0.44-0.64]),lower costs (ratio, 0.68 [95%CI, 0.67-0.69]), and shorter length of stay (ratio, 0.81 [95%CI, 0.79-0.82]). No differences were seen in 30-day all-cause readmission or COPD-specific readmission. |
| Aterbum et al 2015 (42) | Bariatric surgery in patients with severe obesity vs no surgery. | Retrospective cohort study of patients who underwent bariatric surgery in Veterans Affairs (VA) bariatric centers from 2000-2011 (n=9,962). | Cases were matched 1:2 to controls using sequential stratification and an algorithm that included age, sex, geographic region, body mass index, diabetes, and Diagnostic Cost Group.  Kaplan-Meier estimators and stratified, adjusted Cox regression analyses were used to assess outcomes. | All-cause mortality at 14 years. | Adjusted analysis showed no association of bariatric surgery with all-cause mortality in the first year of follow-up, but significantly lower mortality after 1 to 5 years (HR, 0.45 [95%CI, 0.36-0.56]) and 5 to 14 years (HR, 0.47 [95%CI, 0.39-0.58]). |
| Kuramatsu et al 2015 (43) | Oral anticoagulation (OAC) reversal and systolic blood pressure (SBP) control in treating OAC-associated intracerebral hemorrhage (ICH) vs no reversal. | Retrospective cohort study at 19 German tertiary care  centers (2006-2012) n=1176 for analysis of long-term functional outcome, n=853 for analysis of hematoma enlargement. | Multivariable regression  analysis was used to identify predictors of haematoma enlargement. Multivariable regression analysis adjusting for associated co-variates was used to display combined associations of timing and extent of OAC reversal and SBP with hematoma enlargement. Propensity score was calculated from parameters showing statistical associations with OAC resumption. Adjusted Cox regression analyses were performed for the propensity matched cohort of patients with atrial fibrillation. | Frequency of hematoma enlargement in relation to  international normalized ratio (INR) and SBP. In hospital mortality. Incidence analysis of ischemic and hemorrhagic events with or without OAC resumption at 1 year. | Combination of INR reversal <1.3 and SBP <160mmHg at 4 hours was associated with lower rates of hematoma enlargement (18.1% vs 44.2%; OR, 0.28; 95%CI, 0.19-0.42) and lower rates of in-hospital mortality (13.5% vs 20.7%; OR, 0.60; 95%CI, 0.37-0.95). OAC resumption showed fewer ischemic complications (5.2% vs 15.0%) and no significantly increased hemorrhagic complications. Propensity-matched survival analysis in patients with atrial fibrillation who restarted OAC showed decreased HR 0.258 (95%CI, 0.125-0.534) for long-term mortality. |
| Szummer et al 2015 (44) | Fondaparinux vs low molecular weight heparin (LMWH) in patients with non-ST- elevation myocardial infarction (NSTEMI). | Prospective multicenter cohort study from Swedish Web-System for Enhancement and Development of Evidence-Based Care in Heart Disease Evaluated According to Recommended Therapies registry 2006-2010. n= 40,616. | Logistic regression models were used, adjusted for calendar time, admitting hospital, baseline characteristics, and in-hospital revascularization. Sensitivity analysis involved propensity-score matched analysis with exact matching on calendar-time (quarters) and in-hospital PCI. | In-hospital severe bleeding events and death and 30- and 180-day death, MI, stroke, and major bleeding events. | Fondaparinux was associated with reduced in-hospital bleeding events (1.1% vs 1.8%; aOR 0.54; 95%CI, 0.42-0.70) and mortality (2.7% vs 4.0%; aOR 0.75; 95%CI,0.63-0.89). No differences were seen in bleeding events, mortality, recurrent MI or strokes at 30 and 180 days. |
| Jarvik et al 2015 (45) | Early (within 6 weeks) vs late diagnostic imaging (plain films, computed tomography [CT], magnetic resonance imaging [MRI]) of lumbar or thoracic spine in patients with acute back pain without radiculopathy. | Prospective cohort of patients 65 years or older with a new primary care visit for back pain (2011-2013) in 3 US health care systems (n=5,239). | Cases and controls were matched 1:1 using propensity score matching of demographic and clinical characteristics, including diagnosis, pain severity, pain duration, functional status, and prior resource use. | Back or leg pain-related disability measured by the modified Roland-Morris Disability Questionnaire (score range, 0-24; higher  scores indicate greater disability) at 12 months. | No differences were seen between groups in outcomes at 12 months. |
| Foy et al 2015 (46) | Comparisons of effectiveness of 5 testing strategies for patients presenting to emergency departments with chest pain: no non-invasive testing, exercise electrocardiography,  stress echocardiography, myocardial perfusion scintigraphy, or coronary computed tomography angiography. | Retrospective analysis of health insurance claims data  for a national sample of privately insured patients from January 1 to December 31, 2011 (n=421,774). | Multivariate logistic regression analyses were used to examine relationship between the 5 testing groups and downstream interventions, adjusting for potential confounders, including geographic region, age, sex, diabetes, hypertension,  high cholesterol level, ischemic heart disease, inpatient status on admission, and receipt of a non-invasive test within 6 months before the index encounter. | Proportion of patients in each group who received a cardiac catheterization, coronary revascularization procedure, future non-invasive test, acute myocardial infarction (MI) at 7 and 190 days. | No differences were seen between groups in AMI. Compared with no testing, stress echocardiography, exercise electrocardiography, myocardial perfusion scintigraphy, and coronary computed tomography angiography were associated with significantly higher odds of cardiac catheterization (aOR 1.10; 1.63, 2.48, 1.91 respectively) and revascularization procedures (aOR 1.54, 2.41, 2.40, 3.56 respectively) at 7 days. |
| Ornstein et al 2015 (47) | Hospice or non-hospice use in patients receiving palliative care. | Retrospective cohort study using data from the Health and Retirement Study, a nationally representative longitudinal survey of community-dwelling US adults 50 years or older, linked to Medicare claims (n=1016). | Propensity scores were used to control for factors hypothesized to be associated with both likelihood of patient hospice enrolment and spousal depressive symptoms. Sensitivity analyses using multivariate logistic regression analyses were applied to all spouses and subgroup of spouses who were primary caregivers. | Spousal depressive symptom scores measured 0 to 2 years after death with the Center for Epidemiologic Studies Depression Scale, scored from 0 (no symptoms) to 8 (severe symptoms). | No differences were seen in spousal depression symptom scores between decedent groups who were enrolled or not enrolled in hospices. |
| Valley et al 2015 (48) | Admission to intensive care unit (ICU) or general medical ward in older patients with pneumonia. | Retrospective cohort study of Medicare beneficiaries (aged  >64 years) admitted to 2988 acute care hospitals in the United States with pneumonia from 2010 to 2012 (n= 1 112 394). | Linear and logistic regression models were used to adjust for patient and hospital characteristics. Instrumental variable analysis used differential distance to a hospital with high ICU admission (defined as any hospital in the upper 2 quintiles of ICU use) to account for residual confounding. Sensitivity analysis of average treatment effect of ICU admission on 30-day mortality used inverse probability weighting. | All-cause mortality at 30 days. | In adjusted analyses, for the 13% of patients whose ICU admission decision appeared to be discretionary (dependent only on distance), ICU admission was associated with a significantly lower 30-day mortality (14.8% vs 20.5%). |
| Bekelis et al 2016 (49) | Admission to a tertiary stroke centre vs general hospital for patients with acute stroke. | Retrospective cohort study of Medicare beneficiaries  with stroke admitted to a hospital between 2010 and 2013 (n= 865,184). | Multivariate regression analysis attempted to control for all known confounders (age, HCC scores, stroke type, and others). Drive times to tertiary stroke centre were calculated based on zip code centroids and street-level road network data. Instrumental variable analysis based on the differential travel time to tertiary centre was used to control for unmeasured confounding. | Case fatality rates at 7 and 30 days. | Admission to tertiary centre was associated with 1.8% (95%CI, −2.1%to −1.4%) lower 7-day and 1.8% (95%CI, −2.3%to −1.4%) lower 30-day case fatality. Survival benefit at 30 days was seen only for patients traveling less than 90 minutes. |
| Chan et al 2016 (50) | Therapeutic hypothermia in patients suffering in-hospital cardiac arrest. | Prospective cohort study within the national Get With the Guidelines–Resuscitation registry of patients successfully resuscitated from in-hospital cardiac arrest between 2002 and 2014 within 355 US hospitals (n=26,183). | Matched propensity score analysis was performed of all cardiac arrests and separately for non-shockable (asystole and pulseless electrical activity) and shockable (ventricular fibrillation and pulseless ventricular tachycardia) cardiac arrests. | Survival to hospital discharge. Secondary outcome was favourable neurological survival, defined as Cerebral  Performance Category score of 1 or 2 (ie, without severe neurological disability). | Therapeutic hypothermia was associated with lower in-hospital survival (27.4% vs 29.2%; relative risk [RR], 0.88 [95% CI, 0.80 to 0.97] and this result was similar for non-shockable and shockable cardiac arrest rhythms, and was also associated  with lower rates of favourable neurological survival for the overall cohort (17.0% vs 20.5%; RR, 0.79 [95%CI, 0.69 to 0.90]. |
| Gershengorn et al 2016 (51) | Overnight vs daytime extubation in patients receiving mechanical ventilation (MV) in intensive care units (ICUs). | Retrospective cohort study of adults (aged >18 years) in 165 US ICUs enrolled in the Project IMPACT database from 2000 to 2009  (n=97,844). | Multilevel multivariable regression analyses (clustered by individual ICU) identified factors associated with overnight extubation. Propensity-matched pairs were identified who underwent overnight vs daytime extubation (separately for patients with MV duration <12 and ≥12 hours). | Frequency of reintubation in the ICU, ICU and hospital mortality, and ICU and  hospital length of stay (LOS). | For MV duration <12 hours, reintubation rates were similar for both groups (5.9% vs 5.6%). Overnight extubation was associated with: increased mortality (ICU, 5.6%vs 4.6%,p = 0.03; hospital, 8.3%vs 7.0%); shorter ICU LOS (median [interquartile range], 1.1 [0.8-2.3] vs 1.4 [0.9-2.5] days),but no difference was seen for hospital LOS. For MV duration ≥12 hours, overnight extubation was associated with more frequent reintubation in ICU (14.6% vs 12.4%) and higher ICU (11.2%vs 6.1%) and hospital (16.0%vs 11.1%) mortality, but no difference were seen in LOS. |
| Anderson et al 2017 (52) | Tracheal intubation vs no intubation during cardiac arrest. | Prospective cohort study of adult patients suffering in-hospital cardiac arrest in 668 US hospitals from 2000 to 2014 included in the Get With The Guidelines–Resuscitation registry (n= 108,079). | Time-dependent propensity scores were calculated from multiple patient, event, and hospital characteristics where patients intubated at any given minute (from 0-15 minutes) and these were used to match patients at risk of being intubated within the same minute (ie, still receiving resuscitation). | Survival to hospital discharge. Secondary outcomes included return of spontaneous circulation (ROSC) and good functional outcome (defined as a cerebral performance category score of 1 (none or mild disability) or 2 (moderate disability). | Intubation was associated with lower survival (16.3% vs 19.4%; RR = 0.84; 95%CI, 0.81-0.87), lower ROSC (57.8% vs 59.3%; RR = 0.97; 95%CI, 0.96-0.99) and lower frequency of good functional outcome (10.6% vs 13.6%; RR = 0.78; 95%CI, 0.75-0.81). |
| London et al 2017 (53) | Perioperative use of statins vs no statins in patients undergoing major non-cardiac surgery. | Retrospective cohort study of veterans undergoing elective or emergent surgery who were admitted within 7 days of surgery between 2005 and 2010 and sampled by the Veterans Affairs Surgical Quality Improvement Program (VASQIP) (n=180,478). | Propensity score matched analysis was performed using multivariate regression models that included patient, surgery and hospital characteristics. | All-cause 30-day mortality (primary outcome) and  standardized 30-day cardiovascular and non-cardiovascular outcomes captured by VASQIP. | Patients receiving statins had lower 30-day all-cause mortality (RR = 0.82; 95%CI,  0.75-0.89; number needed to treat, 244; 95%CI, 170-432), lower rates of any complications (RR = 0.82; 95%CI, 0.79-0.86; number needed to treat, 67; 95%CI, 55-87) with greatest effect (RR = 0.73; 95%CI, 0.64-0.83) seen for cardiac complications. |
| Xian et al 2017 (54) | Use of antithrombotic treatment vs no treatment in patients with atrial fibrillation (AF) prior to stroke onset. | Retrospective cohort study of  patients with acute ischemic stroke and known history of AF admitted from 2012 to 2015 to 1622 hospitals participating in the Get With the Guidelines–Stroke program (n=94,474). | Multivariable logistic regression models were used to investigate relationships between preceding antithrombotic therapies  with each clinical outcome measure: stroke severity at admission, in-hospital mortality, and modified Rankin score (mRS) at discharge. Analyses were adjusted for baseline demographic and clinical variables prior to the index stroke event, including age, sex, race/ethnicity, insurance, co-morbidities, smoking status, medication use. | Stroke severity as measured by the National Institutes of  Health Stroke Scale (NIHSS; range of 0-42, with a higher score indicating greater stroke severity) and in-hospital mortality. | After adjusting for potential confounders, preceding use of warfarin, new oral anticoagulants, or antiplatelet therapy were associated with lower risk of moderate or severe stroke (aOR = 0.56 [95%CI 0.51-0.60]; 0.65 [0.61-0.71], 0.88 [0.84-0.92], respectively) and in-hospital mortality (aOR 0.75 [0.67-0.85],0.79 [0.72-0.88],0.83 [0.78-0.88] respectively). |
| Axelsson et al 2017 (55) | Use of alendronate vs no alendronate in older patients using oral  prednisolone. | Retrospective cohort study using a national database of patients aged 65 years or older undergoing a health evaluation (baseline) at  Swedish health care facilities who were prescribed alendronate after at least 3 months of oral prednisolone treatment (≥5mg/day) (n = 7,878). | Propensity score matched analysis was performed with multivariate Cox proportional hazards model used to estimate outcomes over time and adjusted for age, sex, weight, height, known fracture-free time, any previous fracture, number of previous fractures, previous hip fracture, previous vertebral fracture, previous fall injury, osteoporosis, Charlson comorbidity index, previous calcium and vitamin D treatment, rheumatoid arthritis, and alcohol-related diseases. | Incident hip fracture at median follow-up 1.3 years. | Alendronate was associated with a lower risk of hip fracture (aHR = 0.35; 95%CI, 0.22-0.54) with no increased risk of upper gastrointestinal tract symptoms or peptic ulcers. No cases of incident drug-induced osteonecrosis and only 1 case of femoral shaft fracture were observed in each group. |
| Haaland et al 2017 (56) | Use of warfarin vs no warfarin in unselected patients as protection against cancer. | Population-based cohort study with subgroup analysis using the Norwegian National Registry coupled with the Norwegian Prescription Database and the Cancer Registry of Norway. Cohort comprised all persons born between 1924 and 1954 and residing in Norway from 2006 to 2012. Cohort was divided into 2 groups—warfarin users and nonusers with persons taking warfarin for atrial fibrillation/ flutter as a subgroup. (n=1,256,725). | Warfarin users were compared with nonusers using incidence rate ratios (IRRs), adjusted for sex and age (2-year age groups). Competing risk regression (method of Fine and Gray) was conducted with death as a competing event. | Incident rate of cancers among patients over 50 years of age. | Warfarin use was associated with significantly lower age- and sex-adjusted IRR in all cancer sites (IRR, 0.84; 95% CI, 0.82-0.86) and in prevalent organ-specific sites (lung, 0.80 [95% CI, 0.75-0.86]; prostate, 0.69 [95% CI, 0.65-0.72]; and breast, 0.90 [95% CI, 0.82-1.00]). Among patients with atrial fibrillation/flutter, the IRR was lower in all cancer sites (IRR, 0.62; 95% CI, 0.59-0.65) and in prevalent sites (lung, 0.39 [95% CI, 0.33-0.46]; prostate, 0.60 [95% CI, 0.55-0.66]; breast, 0.72 [95% CI, 0.59-0.87]; and colon, 0.71 [95% CI, 0.63-0.81]). |
| Presley et al 2018 (57) | Broad-based genomic sequencing (B-BGS) vs routine testing for EGFR mutations and/or ALK rearrangements alone in patients with advanced non-small cell lung cancer (NSCLC) receiving chemotherapy. | Retrospective cohort study of patients with chart-confirmed advanced NSCLC between 2011 2016, and who  received care at one of 191 US oncology practices using the Flatiron Health Database. (n=5,688). | Instrumental variable analysis was performed for 12-month mortality based on the rate of B-BGS (number of patients receiving B-BGS/total number of patients per practice) at the practice where patient received care from the start of first-line treatment. Propensity score–matched survival analysis was performed for overall survival. | Overall mortality at 12 months and overall  survival from start of first-line treatment. | Instrumental variable analysis showed no significant association between B-BGS and 12-month mortality (41.1% vs 44.4%). Propensity score–matched overall survival analysis also showed no difference (42.0% vs 45.1%). |
| Friedman et al 2018 (58) | Surgical left atrial appendage occlusion (S-LAAO) vs no S-LAAO in patients with atrial fibrillation (AF) undergoing concomitant cardiac surgery. | Retrospective cohort study of nationally representative Medicare-linked cohort from the Society of Thoracic Surgeons Adult Cardiac  Surgery Database (2011-2012) of patients aged 65 years and older with AF undergoing cardiac surgery (coronary artery bypass grafting [CABG], mitral valve surgery with or without CABG, or aortic valve surgery with or without CABG) (n=10,524). | Propensity scores with inverse probability–weighted (IPW) Fine-Gray or Cox proportional  hazard models were developed which contained variables reflecting competing risk of death. | Readmission for thromboembolism  (stroke, transient ischemic attack, or systemic embolism) at 3 years. Secondary end points included haemorrhagic stroke, all-cause mortality, composite end point (thromboembolism, haemorrhagic stroke, or all-cause mortality). | IPW analysis showed S-LAAO was associated with lower rate of thromboembolism (aHR 0.67; 95%CI, 0.56-0.81), all-cause mortality (aHR, 0.88; 95%CI, 0.79-0.97), and composite end point (aHR 0.83; 95%CI, 0.76-0.91), lower risk of thromboembolism among patients discharged without anticoagulation (aHR, 0.26; 95%CI, 0.17-0.40). |
| Jakobsen et al 2018 (59) | Bariatric surgery vs specialized medical treatment (including individual or group-based lifestyle intervention programs) in patients with severe obesity (body mass index [BMI] ≥40 or ≥35 and at least 1 comorbidity). | Retrospective registry-based cohort study of consecutive patients with baseline data of exposures from 2005 to 2010 and treated at tertiary care outpatient centre, Vestfold Hospital Trust, Norway, linked with data from Norwegian Prescription Database and Norwegian Patient Registry and local laboratory database (n=1,888). | Binary logistic regressions were applied using mixed models for repeated measures. Possible confounders (age, sex, and BMI at baseline) were included in final models. | Remission and new onset of hypertension based on drugs dispensed; secondary outcomes included changes in comorbidities, adverse events including complications. | Surgically treated patients had greater rate of hypertension remission (31.9 vs 12.4%), and diabetes remission (57.5% vs 14.8%) and lower rate of new-onset hypertension (3.5% vs 12.2%), but increased risk of new-onset depression (8.9% vs 6.5%), treatment initiation with opioids (19.4% vs 15.8%) and need for additional gastrointestinal surgery (31.3% vs 15.5%). |
| Reges et al 2018 (60) | Bariatric surgery (laparoscopic banding,  Roux-en-Y gastric bypass, or laparoscopic sleeve gastrectomy vs usual care obesity management only (provided by a primary care physician and which may include dietary counseling and behaviour modification) in patients with obesity. | Retrospective cohort study in a large Israeli integrated health fund covering 54%of Israeli citizens with less than 1% turnover of members annually (n=33,540). | Each bariatric surgery patient was matched to three nonsurgical  patients according to age group stratified by 5-year intervals, sex, BMI group (stratified from >30 to  >50 in 5-unit intervals) and diagnosis of diabetes. Stratified Cox proportional hazards regression using multiple  matched pairs (3:1) was used to assess association between bariatric surgery and all-cause mortality with adjustment according to age (continuous), SES, population sector, immigrant status, diagnosis of hyperlipidaemia, hypertension, cardiovascular disease, smoking status, level of BMI (continuous) and lipoprotein levels. | All-cause mortality at median 4.3 years follow-up. | Nonsurgical patjents demonstrated higher adjusted mortality than surgical patients (hazard ratio [HR] = 2.02 (95%CI, 1.63-2.52). There was an absolute difference of 2.51 (95%CI, 1.86-3.15) fewer deaths/1000 person-years in surgical vs nonsurgical group. Adjusted HRs were 2.01 (95%CI, 1.50-2.69) for laparoscopic banding, 2.65 (95%CI, 1.55-4.52) for gastric bypass, and 1.60 (95%CI, 1.02-2.51) for laparoscopic sleeve gastrectomy. |
| Bansal et al 2018 (61) | Implantable cardioverter-  defibrillator (ICD) vs no ICD in adults with chronic kidney disease (CKD) and heart failure with reduced left ventricular ejection fraction (HFrEF). | Retrospective cohort study of adults with heart failure and EF of 40% or less and estimated glomerular filtration rate of less than 60 mL/min/1.73 enrolled in 4 Kaiser Permanente health care delivery systems between 2002 and 2012 (n= 5,877). | High-dimensional propensity score based on 500 variables was used to match patients with or without ICD. Cox proportional hazards regression models were used to examine association between receipt of a primary prevention ICD and risk of all-cause death, and a generalized estimating equation Poisson regression was used to examine association of ICD placement with heart failure–related hospitalizations and any-cause hospitalizations. | All-cause death, hospitalizations due to heart failure, and any-cause hospitalizations at mean 3.1 years follow-up. | No difference was seen in all-cause mortality. ICD was associated with increased risk of subsequent HF hospitalization (aRR 1.49; 95%CI, 1.33-1.60) and any-cause hospitalization (aRR 1.25; 95%CI, 1.20-1.30). |
| Berry et al 2018 (62) | Surgical repair of hip fracture vs nonsurgical management in nursing home (NH) residents with advanced dementia. | Retrospective cohort study of NH residents with advanced dementia and hip fracture, but not enrolled in hospice care, using nationwide Medicare claims data linked with Minimum Data Set (MDS) assessments from 2008 to 2013 (n=3,083). | Multivariable Cox proportional hazards regression modelling was performed using propensity score with inverse probability of treatment weighting  (IPTW). | All-cause survival at 6 months. Secondary outcomes comprised documented pain, antipsychotic drug use, physical restraint use, pressure ulcers, and ambulatory status among survivors at 6 months. | Surgery was associated with reduced risk of death of 12% (aHR 0.88; 95%CI, 0.79-0.98). Among 2007 survivors at 6 months, surgery conferred less documented pain (aHR, 0.78; 95%CI, 0.61-0.99) and pressure ulcers (aHR, 0.64; 95%CI, 0.47-0.86). No associations were seen with antipsychotic drug or use of physical restraints. |
| Fisher et al 2018 (63) | Bariatric surgery (Roux-en-Y gastric bypass, sleeve gastrectomy, adjustable gastric banding) vs usual care in severely obese patients with diabetes. | Retrospective cohort study of patients with severe obesity (body mass index [BMI] ≥35) aged 19 to 79 years with diabetes enrolled from 2005 to 2011 in 4 US integrated health systems (n = 20,235). | Multivariable Cox regression analysis was used adjusted for age, race/ethnicity, surgical year, BMI, smoking status, duration of observed diabetes before surgery, insulin use, oral diabetes medication use, uncontrolled blood pressure (≥140 or ≥90 mm Hg), use of angiotensin converting enzyme inhibitor or angiotensin receptor blocker medications, use of any  other antihypertensives, insurance type, estimated glomerular filtration  rate, lipoproteins, statins, peripheral arterial disease, hypertension, and diabetic microvascular disease. | Time to incident macrovascular disease (defined as first occurrence of coronary artery disease  [acute myocardial infarction [AMI], unstable angina, percutaneous coronary intervention, or  coronary artery bypass grafting] or cerebrovascular events [ischemic stroke, hemorrhagic stroke, carotid stenting, or carotid endarterectomy]). | Bariatric surgery was associated  with lower composite incidence of macrovascular events at 5 years (2.1% vs 4.3%; HR 0.60 [95%CI, 0.42-0.86]) and of coronary artery disease (1.6% vs 2.8%; HR 0.64 [95%CI, 0.42-0.99]). No difference was seen in incidence of cerebrovascular disease. |
| Inohara et al 2018 (64) | Renin-angiotensin system (RAS) inhibitor vs no RAS inhibitor prescribed in patients after transcatheter aortic valve replacement (TAVR). | Retrospective cohort study of TAVR procedures performed in the United States (using the Society of Thoracic Surgeons/American College of  Cardiology Transcatheter Valve Therapies Registry) between July 2014 and January 2016 linked to Medicare claims data (n=21,312). | Propensity score (1:1) matching analysis with log-rank test and Cox proportional hazards models were used to evaluate association of prescription of a RAS inhibitor with all-cause mortality. For heart failure readmission, models accounted for competing risk of death. | All-cause death and readmission due to heart failure at 1 year after discharge. Secondary outcomes of health status assessed by Kansas City Cardiomyopathy  Questionnaire (KCCQ). | Patients prescribed RAS  inhibitor had lower mortality rates (12.5%vs 14.9%, HR 0.82 [95%CI, 0.76 to 0.90]), lower heart failure readmission rates (12.0% vs 13.8%; HR 0.86 [95%CI, 0.79 to 0.95]) but no clinically meaningful change in health status. |
| Ray et al 2018 (65) | Proton pump inhibitor (PPI) co-therapy vs no PPI in patients receiving anticoagulants. | Retrospective cohort study of Medicare beneficiaries (data accessed through the Virtual Research Data Center, a cloud-based repository of deidentified Medicare  files) between 2011 and 2015 who had a new prescription for anticoagulants (n= 1,713, 183). | Time-dependent Poisson regression models with 83 study  covariates were fitted to estimate adjusted incidence of  hospitalization for gastrointestinal bleeding .  Sensitivity analysis involved covariate balancing with propensity-score matching exposure groups according to baseline covariates. | Hospitalizations for upper gastrointestinal tract bleeding expressed as adjusted incidence and risk difference (RD) per 10 000 person-years of anticoagulant treatment, incidence rate ratios (IRRs). | Comparing PPI co-therapy vs no co-therapy, the following results were seen: hospitalisations were lower overall (IRR, 0.66 [95%CI 0.62-0.69]), for apixaban (IRR, 0.66 [95%CI, 0.52-0.85]; RD, -24 [95%CI, -38 to -11]), for dabigatran (IRR, 0.49 [95%CI, 0.41-0.59]; RD, -61.1 [95%CI, -74.8 to -47.4]), for rivaroxaban (IRR, 0.75 [95%CI, 0.68-0.84]; RD, -35.5 [95%CI, -48.6 to -22.4]), and for warfarin (IRR, 0.65 [95%CI, 0.62-0.69]; RD, -39.3 [95%CI, -44.5 to -34.2]). |
| Brar et al (66) | Angiotensin-converting enzyme inhibitors (ACEIs) or angiotensin receptor blockers (ARBs) vs no ACEIs/ARBs after hospital discharge in patients with acute kidney injury (AKI). | Retrospective cohort study of data from the Alberta Canada Kidney Disease Network population database of patients with an episode of AKI (defined as a 50% increase between prehospital and peak in-hospital serum creatinine levels) during a hospitalization between 2008 and 2015. (n=46,253). | Propensity scores were used to construct a matched-pairs cohort of patients who did and did not have a prescription for an ACEI or ARB within 6 months after hospital discharge. Multivariable Cox proportional hazards regression models were used to estimate association between use of ACEI or ARB after index hospitalization and all-cause mortality, hospitalization  for renal cause, ESRD, and ESRD or sustained doubling  of serum creatinine concentration. | All-cause mortality at 2 years as primary outcome; secondary outcomes of hospitalization for a renal cause, end-stage renal disease (ESRD), and a composite outcome of ESRD or sustained doubling of serum creatinine concentration. | ACEI or ARB use was associated with lower mortality in patients with AKI after 2 years (adj HR 0.85; 95%CI, 0.81-0.89).  Patients receiving ACEI or ARB had higher risk of hospitalization for a renal cause (adj HR 1.28; 95%CI, 1.12-1.46). No association found between ACEI or ARB use and progression to ESRD. |
| Sheppard et al (67) | Antihypertensive treatment vs no treatment in low-risk patients with mild hypertension. | Data extracted from Clinical Practice Research Datalink, a database of electronic health records from general practices in England, from 1998 to 2015 of patients with mild hypertension (untreated blood pressure 140/90-159/99mmHg) and no previous treatment and no history of cardiovascular disease (CVD) or CVD  risk factors, left ventricular  hypertrophy, atrial fibrillation, diabetes, chronic kidney disease, or family history of premature CVD (n=19,143). | Propensity scores were used to match individuals prescribed or not prescribed antihypertensive treatment using a logistic regression model in which independent variables included CVD risk factors, calendar year of the index date, and the general practice to which patient was registered. Efficacy of antihypertensives was assessed with Cox proportional hazards modelling. | Rates of all-cause mortality, CVD, and adverse events over median 5.4 years follow-up among patients prescribed antihypertensive treatment at baseline. | No association was found between antihypertensive treatment and mortality (HR 1.02; 95%CI, 0.88-1.17) or between antihypertensive treatment and CVD (HR, 1.09; 95%CI 0.95-1.25). Treatment was associated with increased risk of adverse events, including  hypotension (HR 1.69; 95%CI, 1.30-2.20); syncope (HR, 1.28; 95%CI, 1.10-1.50), electrolyte abnormalities (HR, 1.72; 95%C 1.12-2.65), and acute kidney injury (HR, 1.37; 95%CI, 1.00-1.88). |
